# Supplementary material for: Rapidly evolving aphid gall effector proteins exhibit saposin-like folds
Source: bioRxiv. 2026 Mar 28:2026.03.27.712717. Preprint. [Version 1] doi: 10.64898/2026.03.27.712717 (PMC13041983; doi:10.64898/2026.03.27.712717)
Supplement: Supplement 1 [file media-1.pdf]

Supplementary information for Rapidly evolving aphid gall effector proteins exhibit saposin-like folds

Fatema Bhinderwala[1], Aishwarya Korgaonkar[2,3], Kota Gopalakrishna[2,4], Thomas C. Mathers[5,6], Shuji Shigenobu[7], J. Fernando Bazan[8], Saskia A. Hogenhout[5], Guillermo Calero[1], Angela M. Gronenborn[1,*], David L. Stern[2,3,*]

1 Department of Structural Biology, 3501 Fifth Avenue, University of Pittsburgh School of Medicine, Pittsburgh, Pennsylvania, 15261 USA

2 Janelia Research Campus, Howard Hughes Medical Institute, 19700 Helix Drive, Ashburn, Virginia 20147 USA

3 Howard Hughes Medical Institute and Stowers Institute for Medical Research, 1000 E 50th St., Kansas City, Missouri 64110 USA

4 Meso Scale Diagnostics, 16020 Industrial Drive, Gaithersburg, MD 20877 USA

5 Department of Crop Genetics, John Innes Centre, Norwich, NR4 7UH, Great Britain

6 Tree of Life, Wellcome Sanger Institute, Hinxton, Cambridge, CB10 1SA Great Britain

7 Laboratory of Evolutionary Genomics, Trans-Scale Biology Center, National Institute for Basic Biology (NIBB), Nishigonaka 38, Myodaiji, Okazaki 444-8585, Japan

8 ℏ bioconsulting llc, Stillwater, MN 55082 USA

* corresponding authors

ORCID

Fatema Bhinderwala: 0000-0002-3033-8438

Aishwarya Korgaonkar: 0009-0004-4244-0140

Kota Gopalakrishna: 0000-0001-9275-7059

Thomas Mathers : 0000-0002-8637-3515

Shuji Shigenobu: 0000-0003-4640-2323

J. Fernando Bazan : 0000-0002-3645-9935

Saskia Hogenhout : 0000-0003-1371-5606

Guillermo Calero: 0000-0003-3730-4676

Angela Gronenborn: 0000-0001-9072-3525

David L. Stern: 0000-0002-1847-6483

e-mail

Fatema Bhinderwala: fab47@pitt.edu

Aishwarya Korgaonkar: korgaonkara@hhmi.org

Kota Gopalakrishna: kngopala@gmail.com

Thomas Mathers: tm18@sanger.ac.uk

Shuji Shigenobu: shige@nibb.ac.jp

J. Fernando Bazan: jfbazan@gmail.com

Saskia Hogenhout: Saskia.Hogenhout@jic.ac.uk

Guillermo Calero: guc9@pitt.edu

Angela Gronenborn: amg100@pitt.edu

David L. Stern: dstern@stowers.org

Table of Contents:
Supplementary Methods

List of Supplementary Figures:

Figure S1 Mass spectrometry data of recombinant proteins.

Figure S2: Poisson-Boltzmann electrostatics surface of g3873.

Figure S3: Sulfur density in the anomalous diffraction map of g3873.

Figure S4: Possible evolutionary path of the tandem bicycle protein g2703 structure from a helix-swapped permuted form.

Figure S5: Volcano plot of differential expression analysis of genes enriched in salivary glands versus body of gall foundresses of *H. cornu.*

Figure S6: Structural similarity between the N-terminal and C-terminal half of g3873 and saposin-like proteins in the PDB.

Figure S7: Structural similarity between the N-terminal and C-terminal half of g2703 and saposin-like proteins in the PDB.

Figure S8: Structure comparison between CYC domains of G3873 and G2703 to typical Saposins

Figure S9: AF3 and ESMFold predictions for g3873 and g2703 using single amino acid sequences.

Figure S10: AF2 prediction accuracy for all bicycle proteins across seven different aphid species using our custom curated MSA.

Figure S11: AlphaFold2 prediction with custom MSA for G2703 and G3873

Figure S12: Models for several bicycle proteins in ribbon representation from *S. chinensis*.

Figure S13: AF2, AF3 and ESMFold prediction and accuracy (pLDDT scores) for the g107006 tandem bicycle protein.

Figure S14: AF2, AF3 and ESMFold prediction and accuracy (pLDDT scores) for the g6245 tandem bicycle protein

Figure S15: Ribbon models of the medoids representative for each Leiden cluster from Figure 4B.

Figure S16: Species distribution within each Leiden cluster of the t-SNE from Figure 4B.

Figure S17: Bar graphs depicting the proportional contribution of proteins from each species to each Leiden cluster in the t-SNE plot from Figure 4B.

Figure S18: Physicochemical signatures in space filling representations of Leiden cluster medoid models

Figure S19: UMAP of physicochemical signatures for bicycle proteins of different aphid species

Figure S20: Bar graphs depicting the proportional contribution of proteins from each species to each Leiden cluster in the UMAP plot from Figure 5.

Figure S21: Violin plots of physicochemical properties by cluster and species.

Figure S22: Shannon entropy of bicycle protein diversity mapped onto a ribbon representation of g3873.

List of Supplementary Tables:

Table S1: Expression and crystallization results for bicycle genes tested for recombinant protein expression

Table S2: Crystal data collection parameters and structure statistics for g2703 and g3873.

Table S3: Disulfide bond conformations in the X-ray structure of G3873.

Table S4: Foldseek hits for g3873 and g2703 identify only poor quality matches (.xlsx file).

Table S5: Cα RMSD values between all known saposin structures in the PDB and the saposin-like domains in G3873

Table S6: TM scores for all known saposin-like proteins in the PDB and the saposin-like domains in G2703

Table S7: Statistics of AF2-predicted models for all seven aphid species

Table S8: List of physicochemical features

Table S9: Physicochemical properties for 2400 Bicycle proteins

Table S10: Metrics of structural space

Table S11: Genome sequencing statistics

Supplementary Methods:

Materials: All general chemicals were purchased from Fisher Scientific. Selenomethionine for labeling was purchased from Millipore Sigma and all crystallography screens and materials to prepare custom crystallization solutions were purchased from Hampton Research.

Mass spectrometry for recombinantly expressed proteins:

All ESI LC-MS measurements were performed at a protein concentration of 1 μM on a Bruker Q-TOF instrument, using a reverse-phase AdvanceBio peptide guard column (Agilent Technologies), with mobile phases A and B comprising 5% acetonitrile with 0.01% FA and 80% acetonitrile with 0.01% FA, respectively. The resulting LC-MS spectra were processed using Bruker Compass Software, and the MS data were processed using Maximum Entropy-based deconvolution to obtain the M+ ion mass for each sample. The instrument was calibrated using the ESI Low Tuning mix I (Agilent Technology) to a 1.0 ppm mass % difference before each use.

Ellman's assay to determine free sulfhydryl groups in G3873

Purified protein was buffer exchanged into 0.1 M sodium phosphate buffer, pH 8, 1 mM EDTA and concentrated to 120 μM prior to use in the Ellman's assay. A working solution of Ellman's reagent (DTNB) at 10 mM concentration in 0.1M sodium phosphate buffer, pH 8, was freshly prepared. A six-point calibration curve was prepared using reduced L-glutathione and L-cysteine over a linear range from 10-200 μM of total thiol. Assays were performed in a 96-well plate format by mixing 5 μL of sample with 95 μL of DTNB working

solution (three technical replicates per condition). Plates were incubated at room temperature for 10 min, and absorbance at 412 nm was measured using a Tecan Spark spectrophotometer. Total free sulfhydryls were calculated using Beer-Lambert's law using the molar extinction coefficient of DTNB of 14,150 M^{-1} cm^{-1}.

Calculation of disulfide bond conformation and bond energies in G3873

Disulfide-bond geometry was analyzed with the UCLA Disulfide Bond Dihedral Angle Energy Server by submitting the PDB coordinates of the experimental structure of G3873. For each disulfide linkage, the server then calculated the five defining cystine dihedral angles, $\chi1$, $\chi1'$, $\chi2$, $\chi2'$, and $\chi3$, and the corresponding empirical dihedral energy (1). Dihedral energy was calculated in kJ/mol using the following equation:

$$E = 8.37(1 + \cos 3\chi1) + 8.37(1 + \cos 3\chi1') + 4.18(1 + \cos 3\chi2)$$
$$+ 4.18(1 + \cos 3\chi2') + 14.64(1 + \cos 2\chi3) + 2.51(1 + \cos 3\chi3)$$

The resulting dihedral angles and energies for each disulfide bond are reported in Table S3 and were used to assess relative torsional strain and conformational favorability within the structure.

AlphaFold3 and ESMFold predictions for bicycle proteins:

Protein models were generated using the respective web servers for AF3 (2) and ESMFold (3). For each protein of interest, the corresponding amino acid sequence was submitted to the server using default settings. Predicted models were downloaded, and the respective per-residue confidence scores (pLDDT) were retained as B-factor. The top-ranked model (as returned by the server) was used for comparisons throughout.

Calculation of backbone RMSD values and TM-score between the X-ray structures of G3873 and G2703 and all known saposin fold proteins in the PDB:

Structural similarity between the N- and C-terminal CYC domains of G3873 and G2703 and all 52 PDB deposits with at least one saposin-like domain (classified by InterPro)(4) was assessed for individual chains. Each structure was compared to G3873 and G2703 by running TM-align for every pair. The TM-scores reported by TM-align (5) between the two structures were normalized to the length of the target protein. For each deposit-target pair, backbone RMSD was also computed.

Shannon's entropy and sequence conservation

Sequence diversity was quantified using Shannon entropy computed from a MSA. For each alignment column, the amino acid frequency was calculated across all sequences and converted into a per-position Shannon entropy score. Protein sequences were first filtered by length, and sequences ranging from 120–300 were retained. An MSA was generated from the filtered sequences using MAFFT. Gaps were excluded from frequency calculations, and positions with insufficient coverage were omitted from the summary statistics.

Shannon entropy per MSA column (bits), computed over 20 amino acids only (gaps ignored).

Formula:

$$H_j = - \sum_{a \in AA20} p_j(a) log_2 \, p_j(a)$$

Where: $p_j(a) = n_j(a)/N_j$, with N_j = number of non-gap AA 20 characters in column j.

The first sequence in the alignment was used as the reference to map column entropies onto residue positions. To visualize entropy on the structures, the reference sequence was globally aligned to G3873, and entropy values were written into the B-factor field of matched residues.

Heatmaps and dendrograms for ABEGO Levenshtein distances for bicycle proteins

Sequence collection and preprocessing: Protein sequences were compiled into a FASTA file (n=2403) with identifiers encoding protein name and length (e.g., hc_g3873|chain=A|len=169). All sequences with >0.99 sequence identity were removed from downstream analyses.

ABEGO string generation: ABEGO is a 5-letter code for protein backbone conformation that bins backbone dihedral angles ϕ and ψ for each residue into a small number of common regions in the Ramachandran map (6): A corresponds to the right-handed α helical basin, B and E correspond to the extended/β strand-like basin, G corresponds to the left-handed α helical basin favored by glycine residues, and O captures outlier conformations. We used the following implementation: G (αL/left-handed region; $0 \leq \phi \leq 120$ and $-60 \leq \psi \leq 90$), A (αR/right-handed region; $-120 \leq \phi \leq -20$ and $-90 \leq \psi \leq 50$); B (β region; $-180 \leq$

$\phi \leq -90$ and $\psi \geq 90$ or $\psi \leq -120$), E (extended region; $-180 \leq \phi \leq -60$ and $50 \leq \psi \leq 180$), and O (other/undefined: all remaining (ψ) combinations.

Visualization: For each distance matrix, heatmaps were generated using a continuous color map with low values indicating greater similarity and high values indicate greater dissimilarity. The lower bound of the color maps was changed to enhance contrast among intermediate distances, depending on whether the raw or ABEGO distances are displayed. Hierarchical clustering was performed using average-linked agglomeration applied to pairwise distances, and the lowest tiers of the dendrogram were colored to correspond to the species in which the genes were found.

Calculating and mapping physicochemical properties

A total of 22 physicochemical features were computed for each protein model (Table S7). These properties combine both sequence-derived and structure-derived properties that capture composition, hydrophobicity, electrostatics, and amphipathic character. Solvent-accessible surface area (SASA) was computed using FreeSASA (7).

Atom-level SASA values were combined to yield residue-level SASA and summed across all residues to obtain the total SASA. Residues were assigned to three classes: hydrophobic, positively charged, and negatively charged, and SASA values were summed within each class to obtain SASA_hydrophobic, SASA_pos and SASA_neg. The hydrophobic surface fraction (a potentially relevant property that can regulate saposin-like activity and protein-protein interaction) was reported as a frac_sasa_hydrophobic. In each case, the residue was considered surface-exposed for these calculations if the SASA was >5Å.

Surface hydropathy: Surface hydropathy was quantified using the Kyte-Doolittle scale, and two properties were calculated: a SASA-weighted mean KD value and a median KD value over the exposed residues.

Surface roughness: A global proxy of surface roughness was computed as the ratio between total SASA to the surface area of the 3D convex hull using the SciPy (8) ConvexHull function.

Surface hydrophobic patchiness: Hydrophobic patches were identified by clustering the exposed hydrophobic residues using sidechain centroid coordinates and the Density-based special clustering of applications of noise (DBSCAN) (9). This yields three parameters: the number of hydropatches, the mean size of the patch, and the maximum size of the patch.

Charge and charge-density: Sequence level composition was computed as the fraction of positively charged, negatively charged, and hydrophobic (FLIV) residues. This was combined with net charge, exposed net charge, and exposed net charge over SASA.

Surface charge dipole: To capture spatial polarization of surface charge, a SASA-weighted dipole vector was computed from exposed charge residues.

Amphipathic helical content: The maximum hydrophobic moment was computed as the largest hydrophobic moment value observed over a helix, and an amphipathic helical fraction was calculated as a hydrophobic moment > 0.35 over a helix.

TM-scores between AF2-predicted models of bicycle proteins from Hormaphis cornu and the X-ray structures of G3873 and G2703

The TM-scores between all reliably predicted (median pLDDT >60 over 80% of the residues) bicycle proteins in *Hormaphis cornu* (n=243, for sequence lengths of 120-300 residues) were computed using the experimental X-ray structures of G3873 and G2703 as

targets. The resulting TM-scores were visualized as scatter plots to illustrate the structural diversity in the set.

All-vs-all TM-score matrix for all reliable AF2-predicted models of bicycle proteins of seven aphid species

An all-vs-all TM-score matrix using the TM-align tool was calculated from 2400 models, running TM-align for each pair (i, j). Two TM-scores are obtained for each comparison, one normalized by the length of each of the two polypeptide chains (i and j). The TM-scores were converted into a distance matrix, with structurally similar proteins exhibiting small distances (high TM-scores). A kNN graph constructed from the TM-score-derived distances connects each protein to its nearest neighbor. Community grouping was performed using the weighted KNN graph using the Leiden algorithm (10). The resulting structural similarity space was embedded into two dimensions using t-SNE (11)

Medoid selection in physicochemical and the structural space

For each Leiden cluster C, a representative medoid structure was selected as the member with minimal mean distance to all other members of the same cluster:

$$m = arg\ min_{i \in C} \frac{1}{\mid C \mid -1} \sum_{j \in C, j \neq i} D_{ij}$$

The medoid maximizes the average TM-score compared to other cluster members. Structural models of medoids were used as representatives for cluster-specific visualization.

APBS electrostatic surfaces

Electrostatic surfaces were generated for AF2-predicted medoid models. For each medoid coordinate set, atomic charges and radii were assigned using pdb2pqr. Protonation states were assigned at pH 7.0 using the AMBER forcefield and outputs written as .pqr files for APSB input. Electrostatic potentials were computed using APBS (12, 13) using the linearized Poisson-Boltzmann equation, a protein dielectric of 2.0 and a solvent dielectric of 78.0. The solvent probe radius was set to 1.4 Å, the surface density was set to 10.0, and the ionic concentration was set to 0.15 M of monovalent ions. Electrostatic maps were written as .dx grids and used to generate structure frames using a fixed color ramp of −5 to +5 kT/e in PyMOL (14).

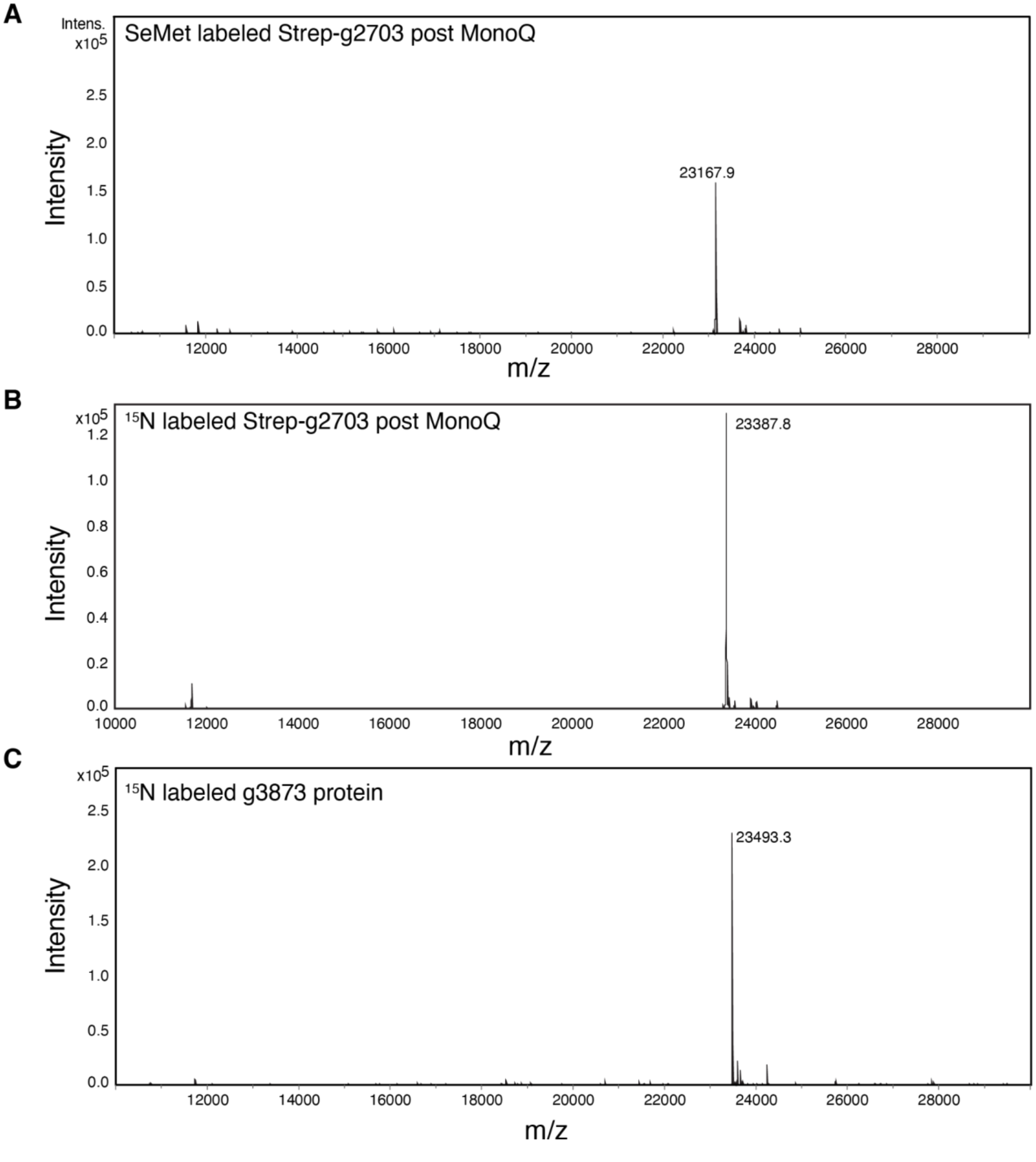

Figure S1: (A and B) ESI Mass spectrometry data of selenomethionine (SeMet) labeled Strep-tagged G2703 and 15N-labeled strep-tagged G2703 after cleavage of the tag. The calculated predicted masses for the full-length protein without the C-terminal 10x His-tag is 23387 Da. The predicted calculated mass is 23169 Da for the protein with a single SeMet residue. (C) ESI Mass spectrometry data of cleaved ^{15}N-labeled G3873 protein. The predicted calculated mass is 23490 Da.

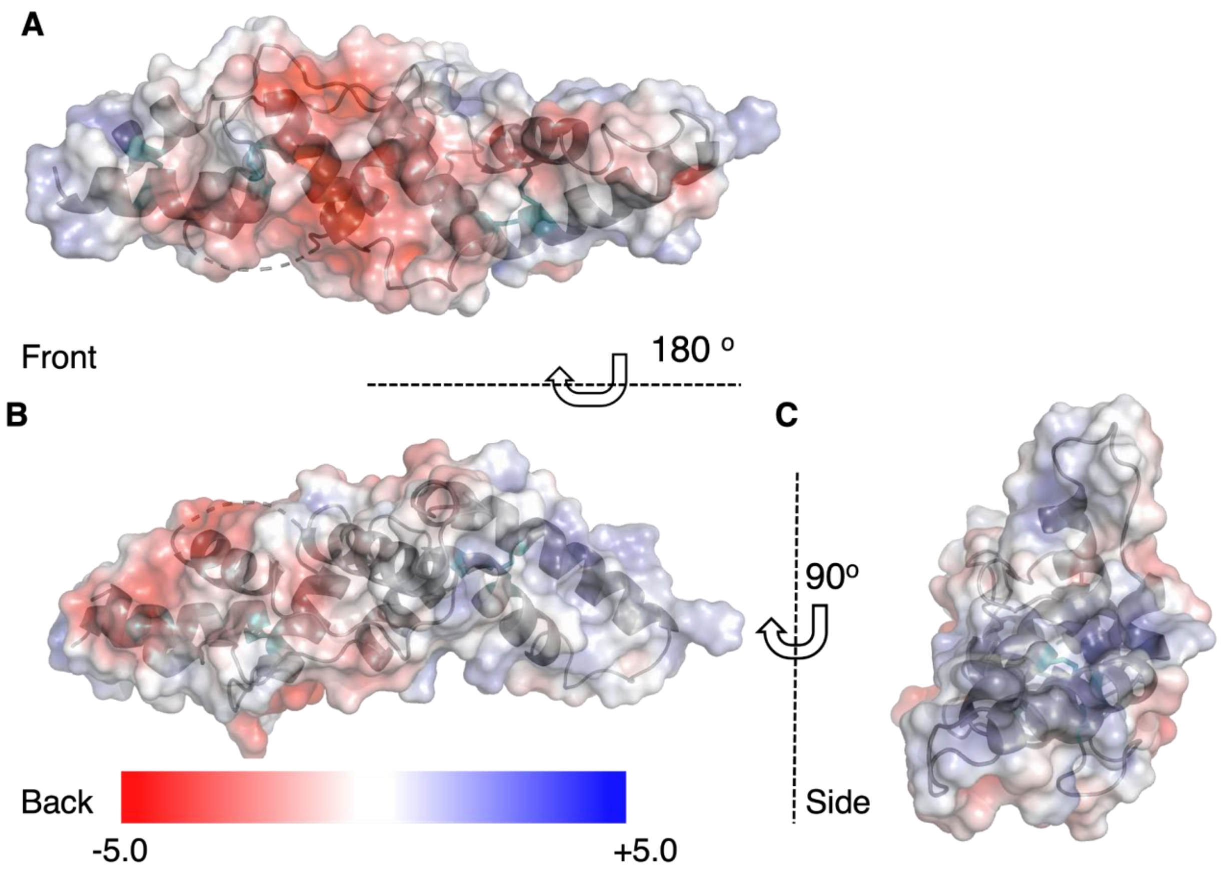

Figure S2: (A) Front, (B) back, and (C) side view of G3873 in space filling representation (gray) with the Poisson-Boltzmann electrostatic surface colored from red to blue for -5.0 to +5.0.

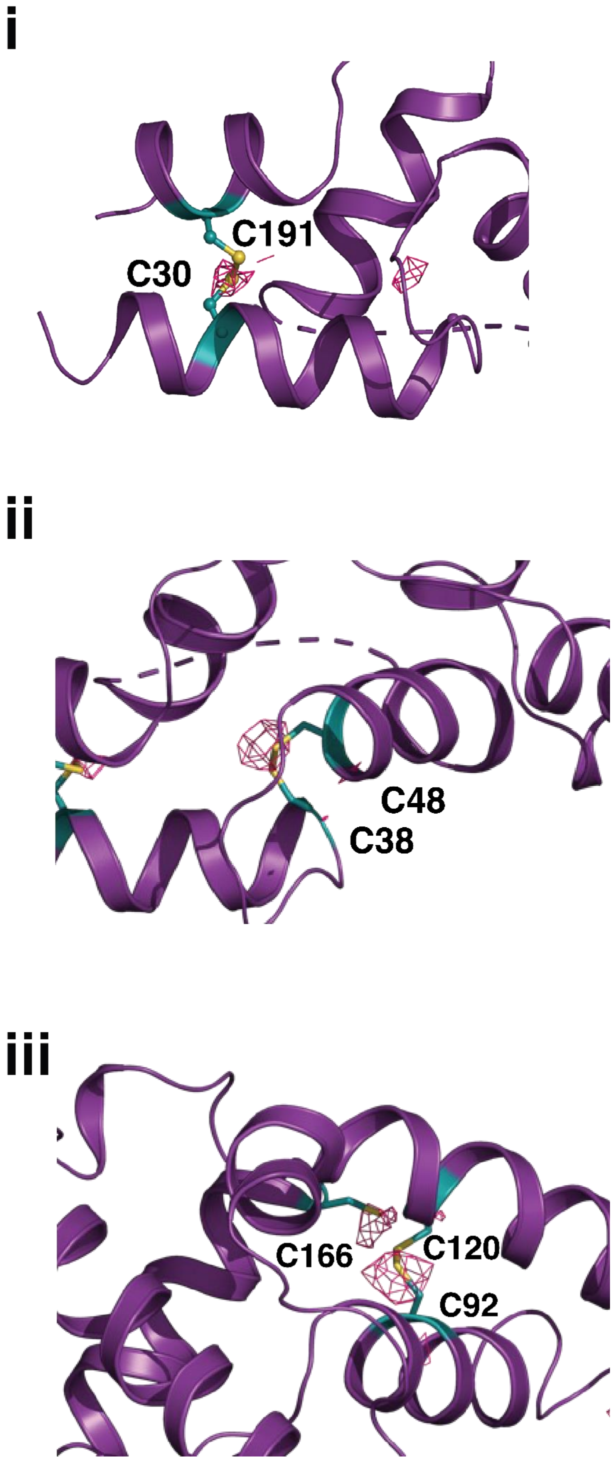

Figure S3: Sulfur density in anomalous diffraction map (magenta) at each of the three disulfide bonds (cyan and yellow ball and stick representation) in G3873 (purple ribbon representation) for (i) C30-C191, (ii) C38-C48, and (iii) C92-C120.

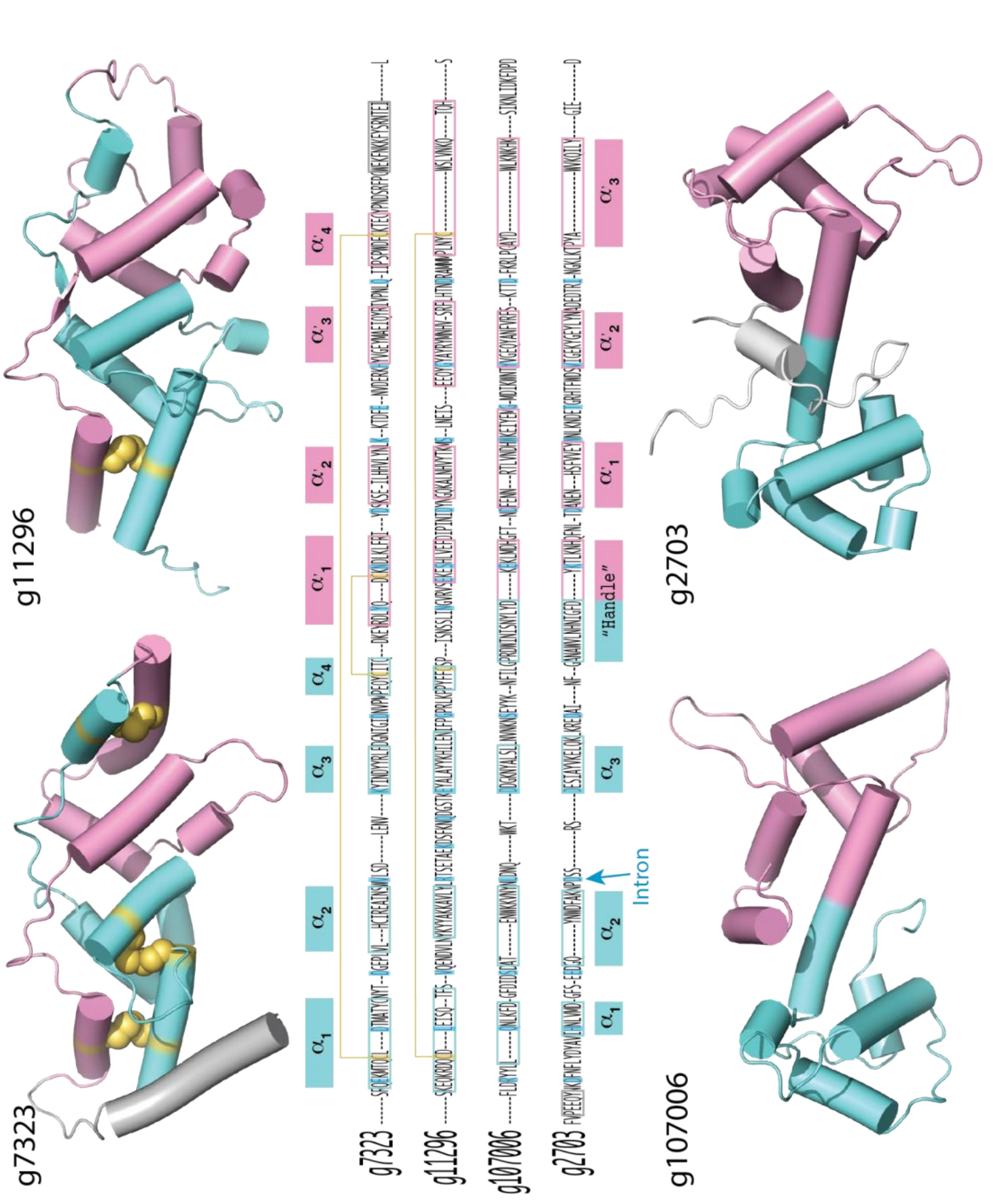

Figure S4: The central "fusion helix" of g2703 may have evolved from the α_4 and α'_1 helices of permuted bicycle proteins. Multiple sequence alignment (MSA) of g2703 and three selected paralogs of g2703 is shown in the middle of the figure. Proteins were selected to illustrate a hypothetical evolutionary path from a permuted protein with two disulfide bonds (g7323), through a protein with one disulfide bond (g11296), to two non-permuted proteins (g107006 and g2703). AF2 predicted structures are shown above and below the MSA in cylinder representation. Within the MSA, secondary structure elements are shown in boxes, disulfide bands as yellow connections, and amino acids that sit at intron positions are highlighted in blue. The positions of the helices are labeled above and below the MSA as α_1, α_2, α_3, α'_4 (colored in aqua) and α'_1, α'_2, α'_3, α_4 (colored in pink) with the continuous 'fusion' helix in G2703, and G107006 colored both aqua and pink. Cysteine residues are

highlighted yellow. The MSA was generated using an intron-aware alignment program (15), because there is insufficient sequence similarity between the proteins to correctly estimate the homologous locations of helices from sequence alone.

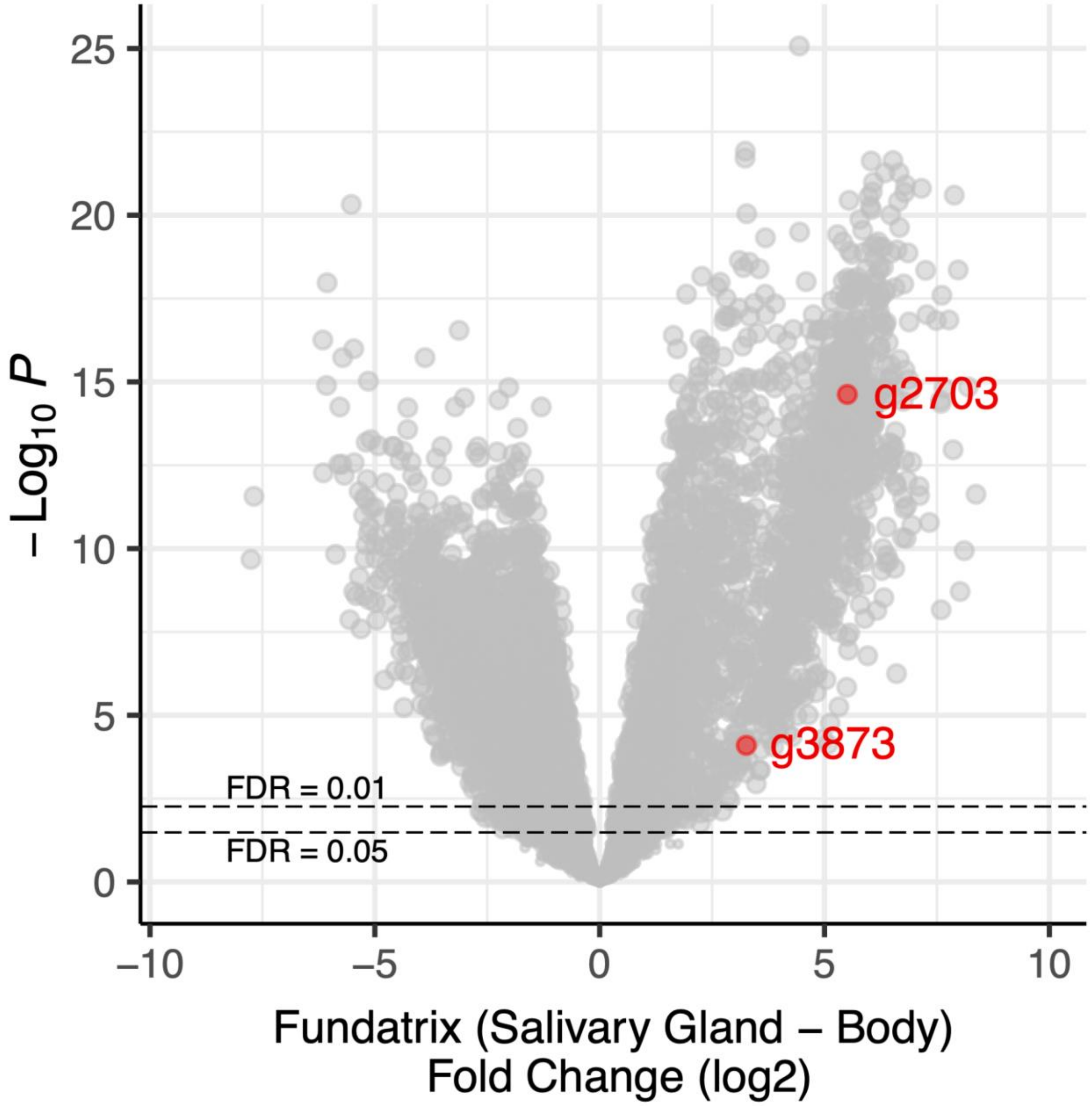

Figure S5: Volcano plot of differential expression analysis of genes enriched in salivary glands versus body of gall foundresses of *H. cornu.* The genes *g2703* and *g3873* are highlighted. The data and analysis pipeline are found in publication (16).

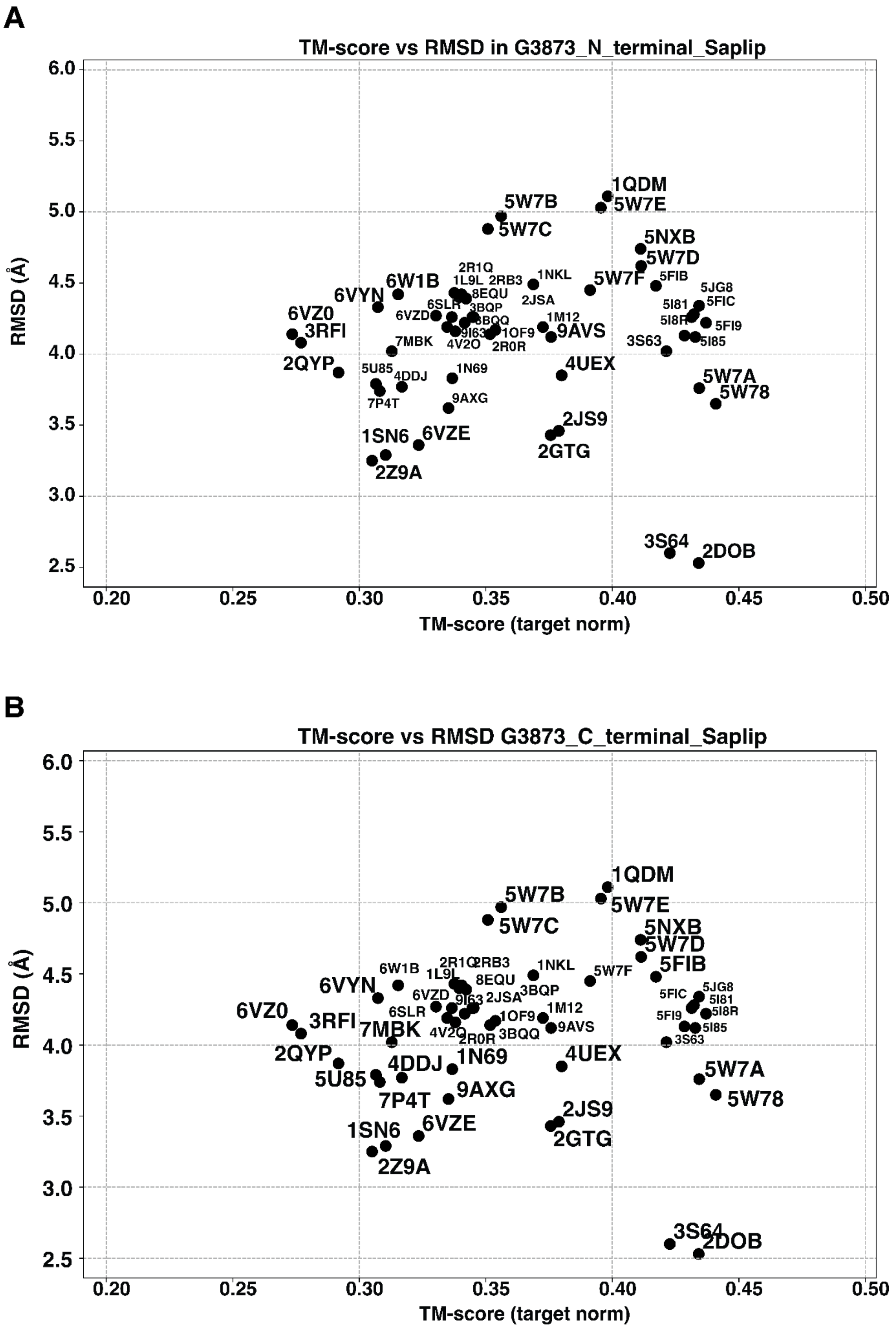

Figure S6: Plots of RMSD values for the saposin N-terminal (A) and C-terminal (B) CYC motif domains of G3873 versus saposin-like proteins in the PDB.

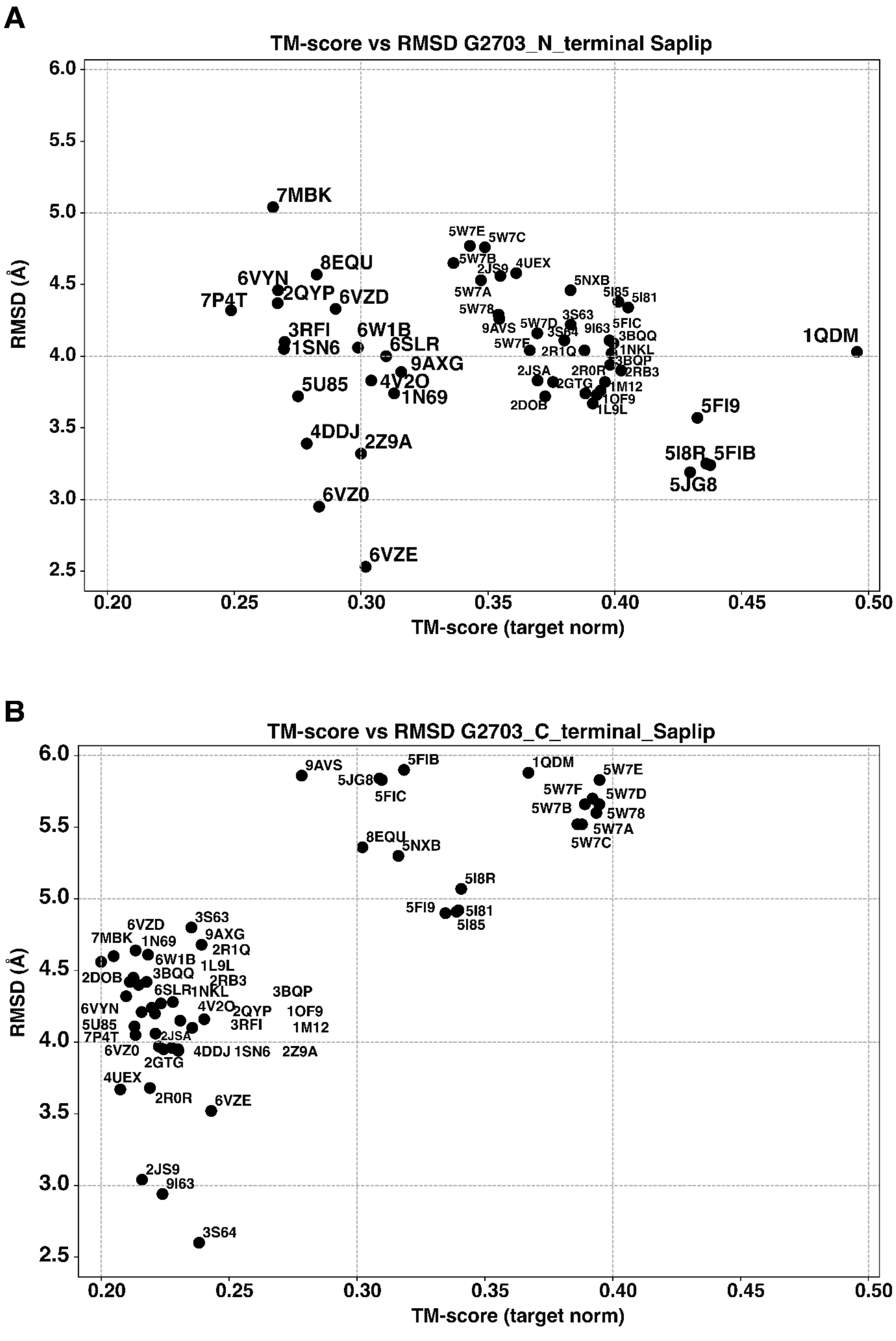

Figure S7: Plots of RMSD values for the saposin N-terminal (A) and C-terminal (B) CYC motif domains of G2703 versus saposin-like proteins in the PDB.

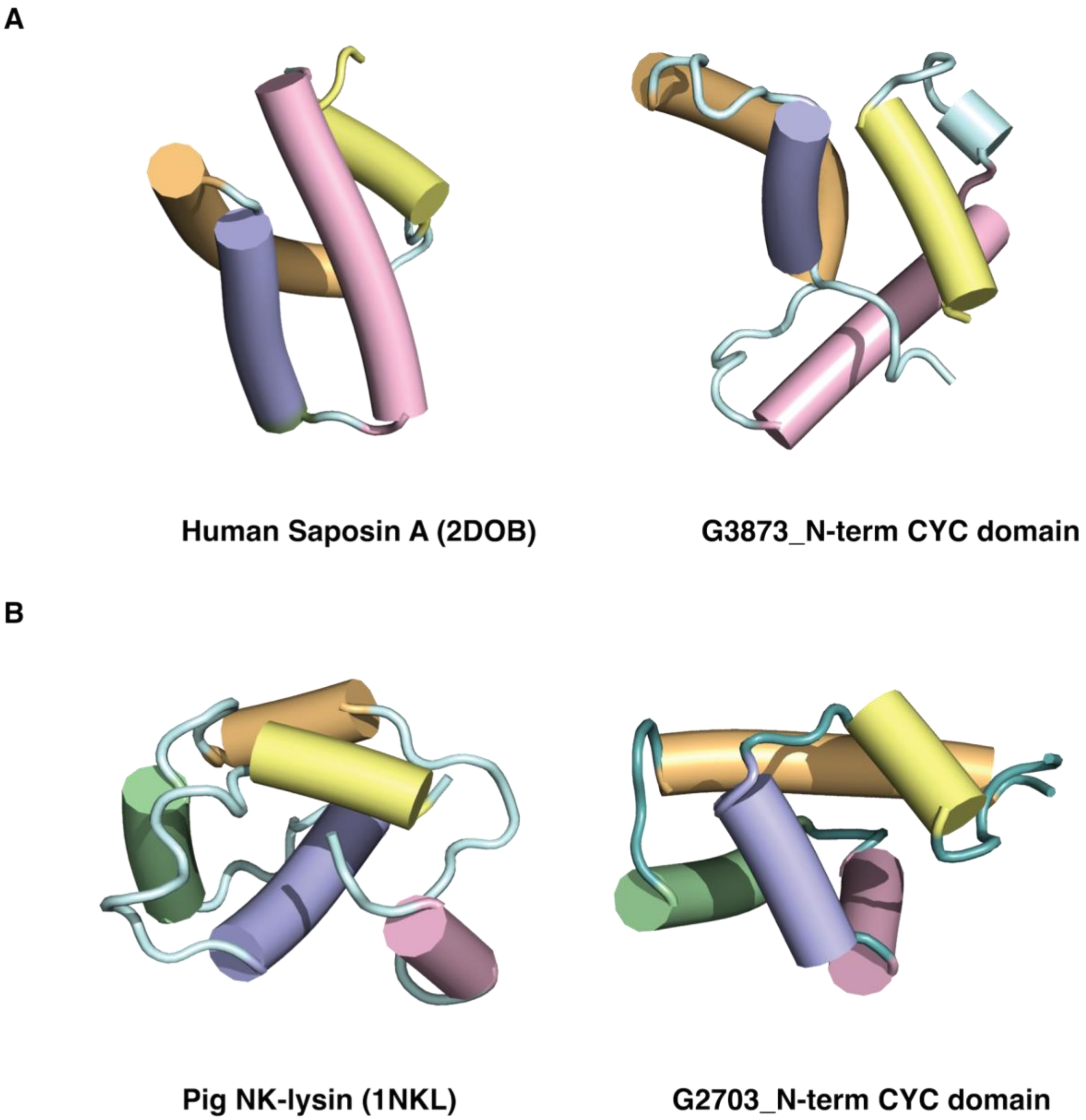

Figure S8: Structure comparison between the four-helix bundles of saposin-like proteins and bicycles CYC domains. (A) N-terminal domain of g3873 next to human saposin A (PDB ID: 2DOB). (B) N-terminal domain of g2703 next to pig NK-lysin (PDB ID: 1NKL). Helices are represented as cylinders and colored to show structural similarities.

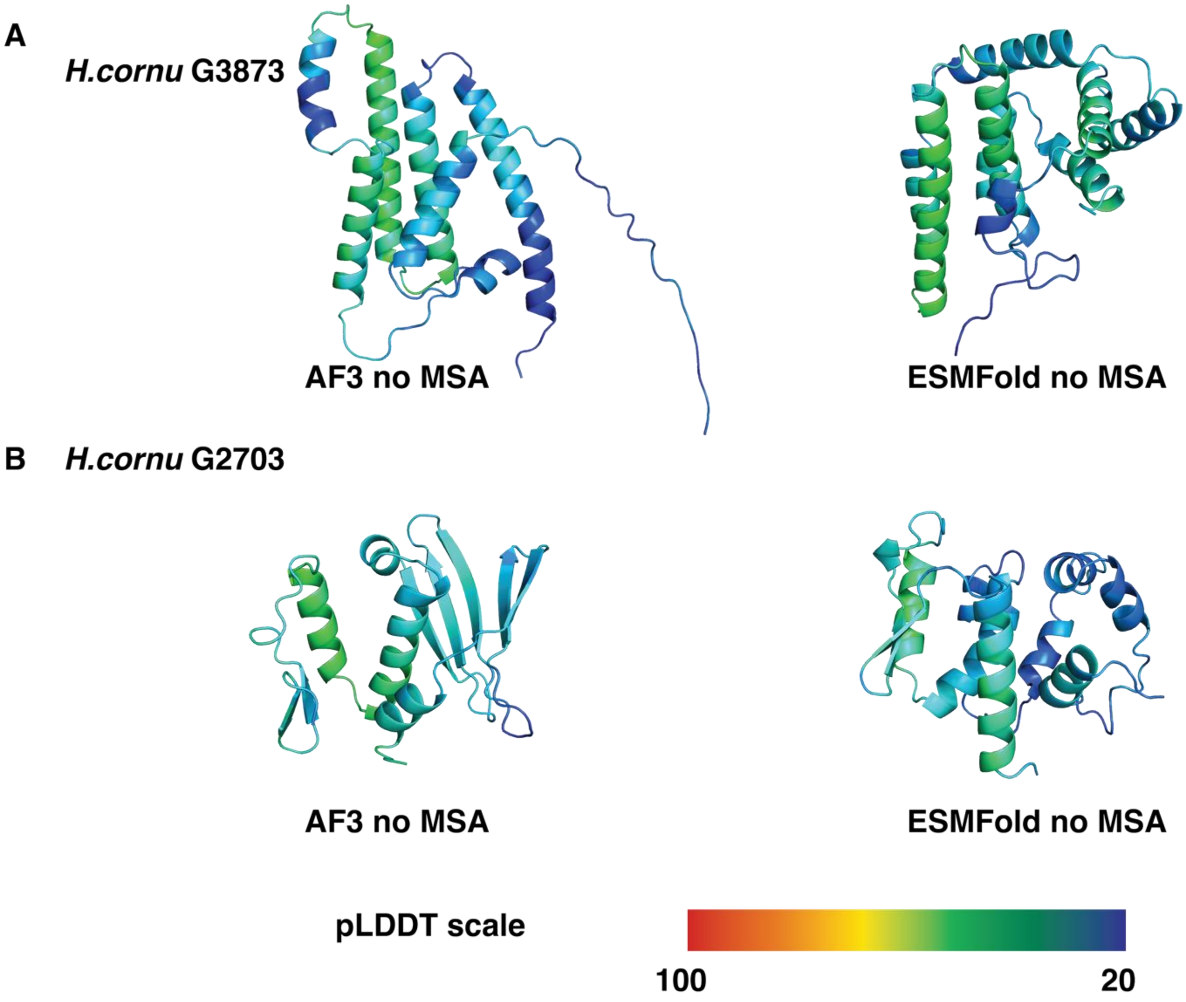

Figure S9: AF3 and ESMFold predicted models for G3873 (A) and G2703 (B) using a single amino acid sequence. The ribbon diagrams are colored according to their pLDDT scores (bottom bar).

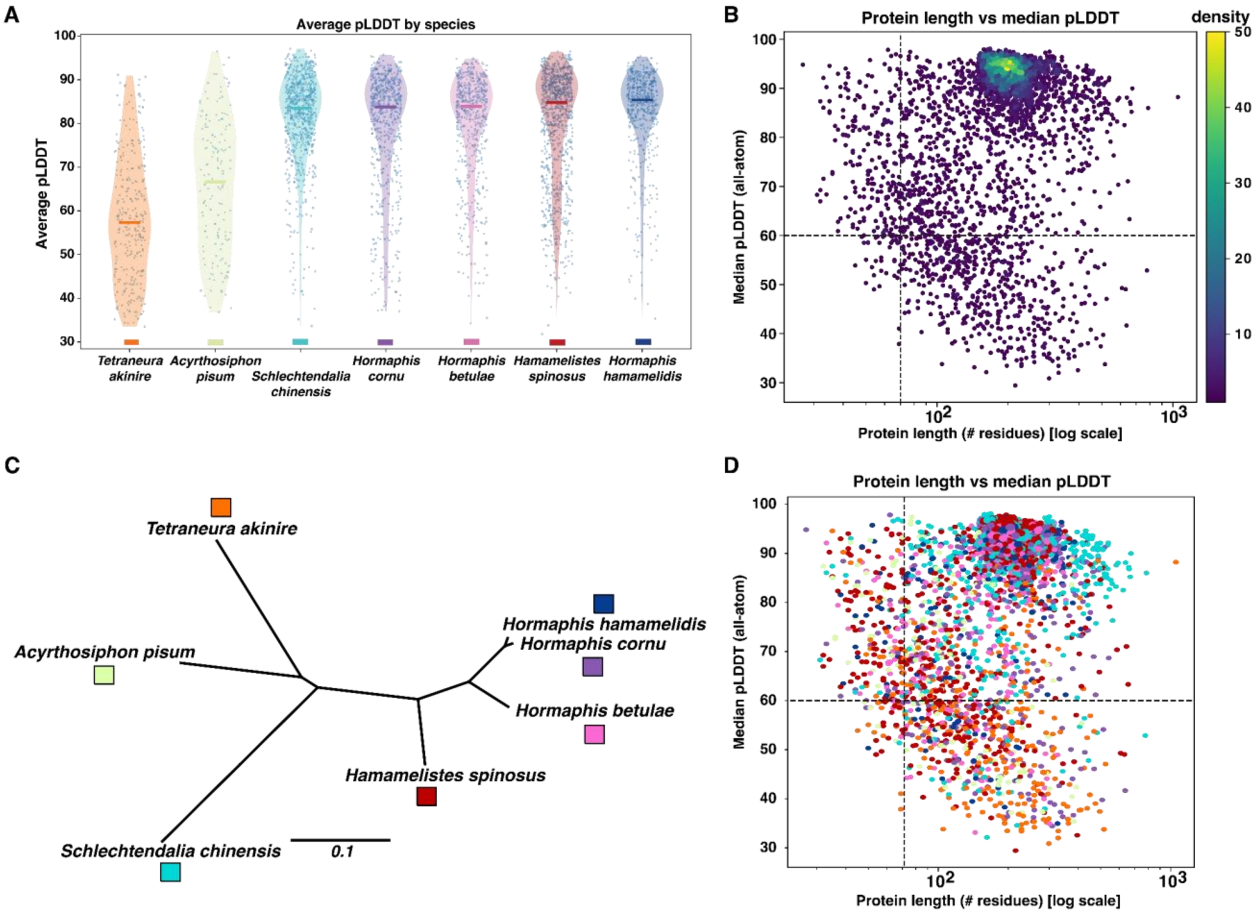

Figure S10: Distribution of pLDDT values of AF2-predicted models (A) for each aphid species and (B) versus protein length colored by density and (D) by aphid species (key in color squares adjacent to each species in an unrooted phylogeny in (C)). Phylogeny branch lengths are proportional to the number of substitutions per site in all concatenated conserved proteins (17) and the scale bar represents 0.1 substitutions per site.

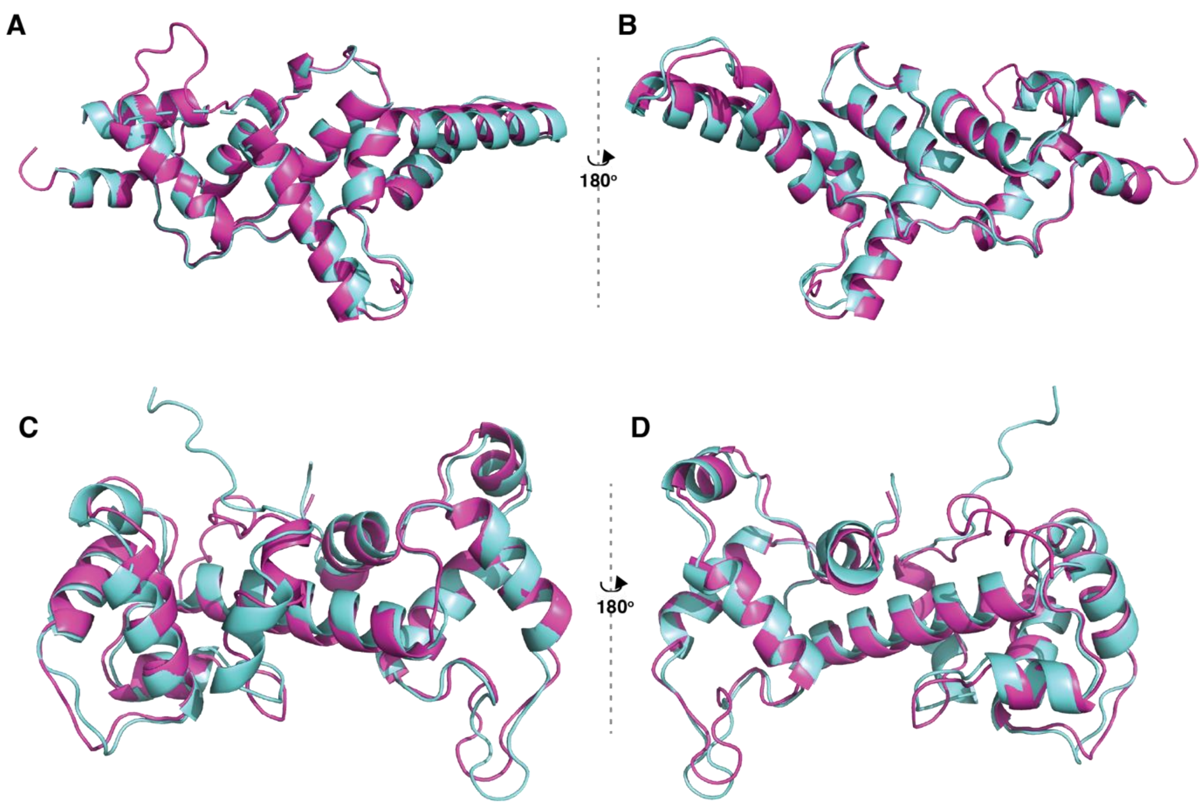

Figure S11: AF2-predicted models (cyan) of (A, B) g3873 and (C, D) g2703 using custom MSAs superimposed on their respective crystal structures (magenta).

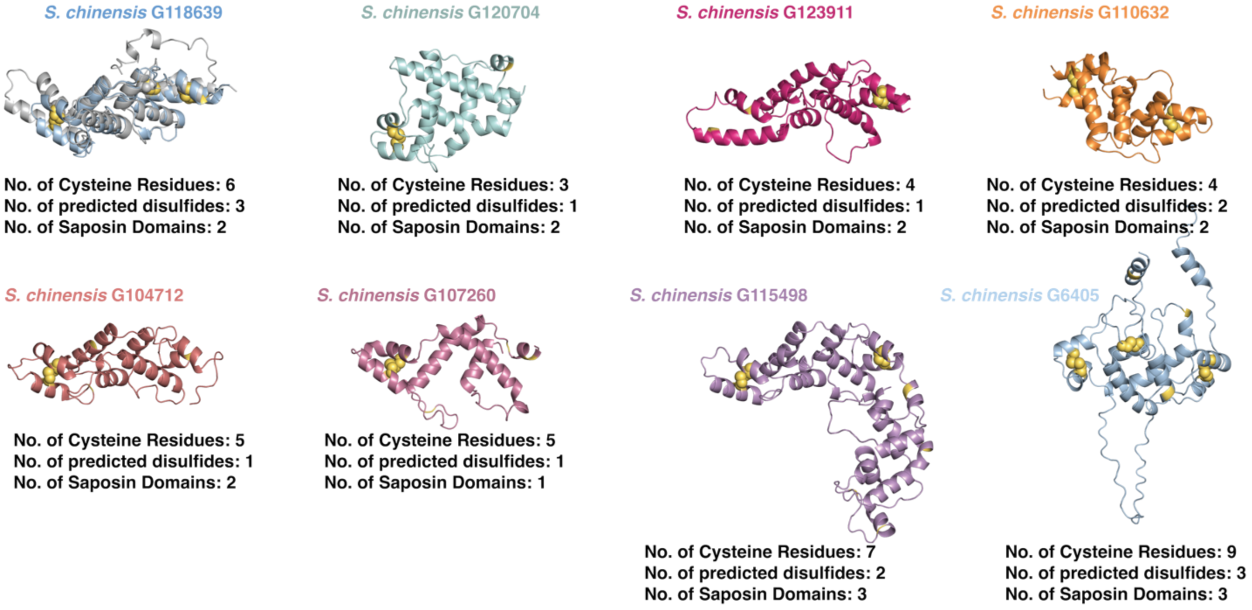

Figure S12: AF2-predicted Bicycle protein models from *Schlechtendalia chinensis* in ribbon representation. At the top left, the g119639 model (blue) is superimposed on the X-ray structures of *H. cornu* g3873 (gray). Cysteine residues are shown as yellow spheres.

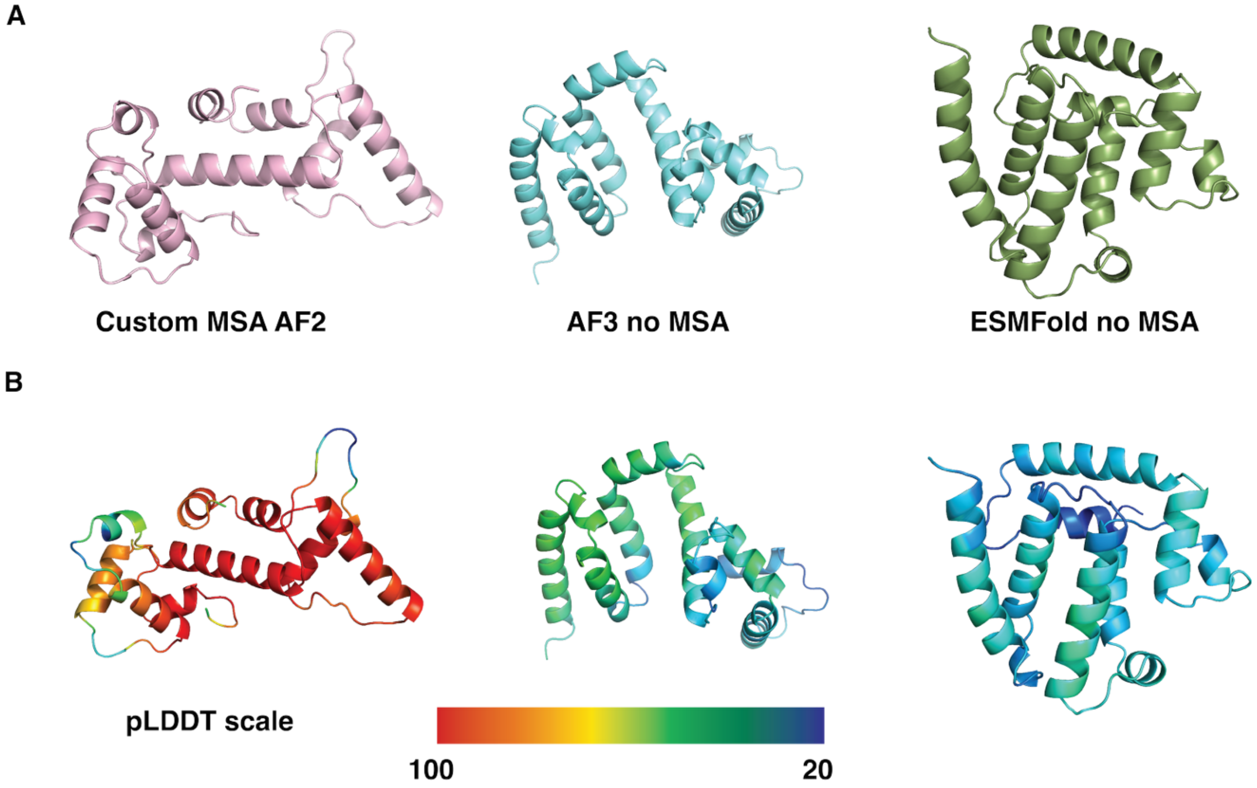

Figure S13: (A) Predicted models of g107006 estimated using AF2 with a custom MSA (pink), AF3 without an MSA(aqua), and ESMFold without an MSA (green) in ribbon representation. (B) The models shown above in (A) colored by pLDDT scores ranging from 20 (blue) to100 (red).

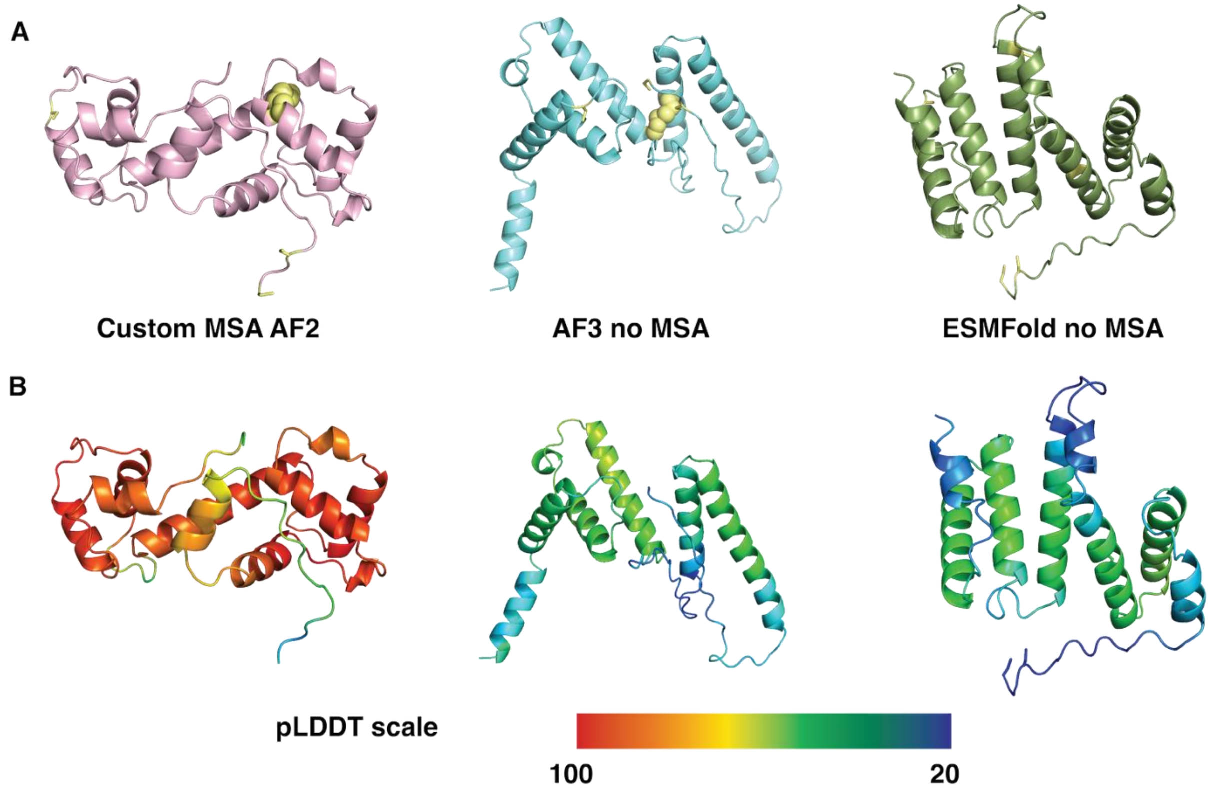

Figure S14: (A) Predicted models of g6245 estimated using AF2 with a custom MSA (pink), AF3 without an MSA(aqua), and ESMFold without an MSA (green) in ribbon representation. (B) The models shown above in (A) colored by pLDDT scores ranging from 20 (blue) to100 (red).

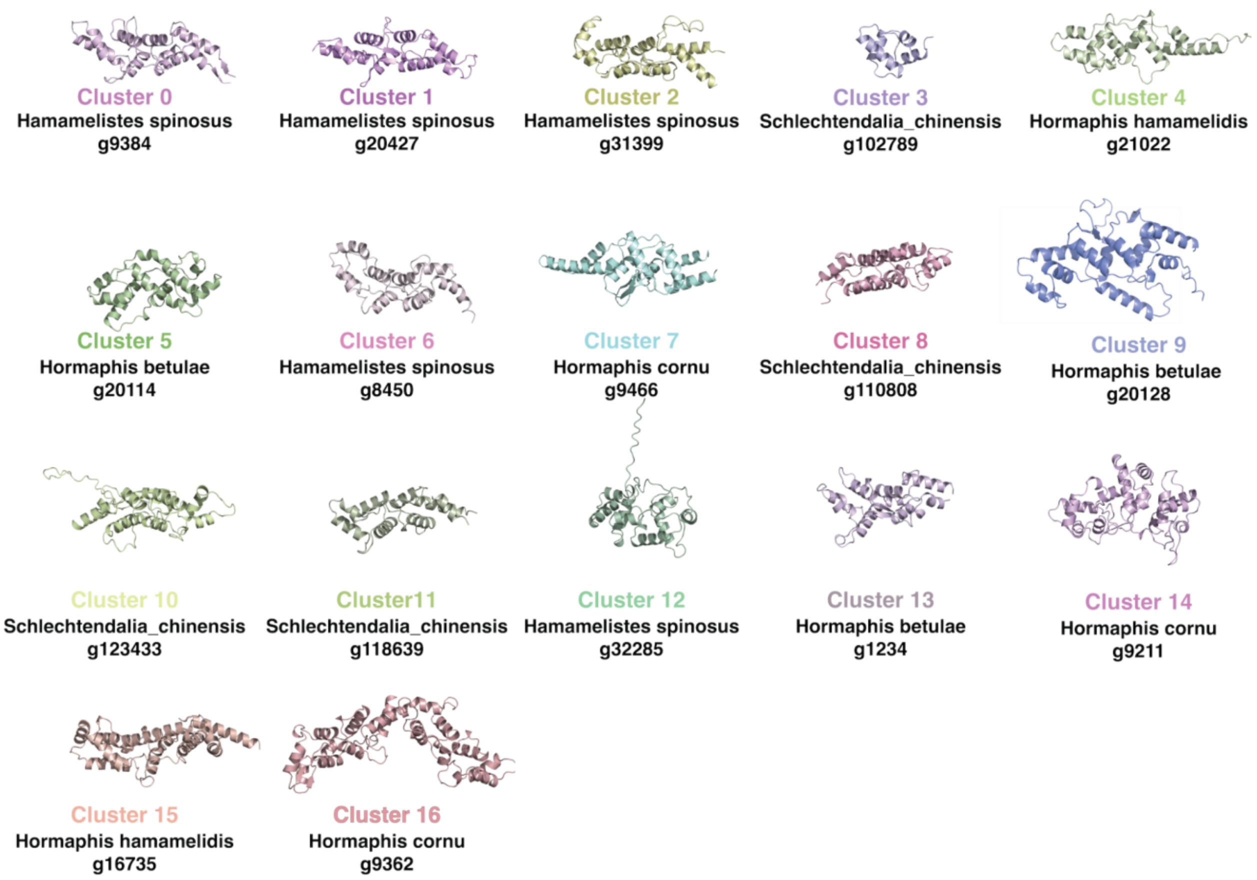

Figure S15: Ribbon models of the medoids representative for each Leiden cluster from Figure 4B shown in their corresponding cluster colors. The medoid possesses the highest average TM-score (the lowest average distance) to all other members of that cluster.

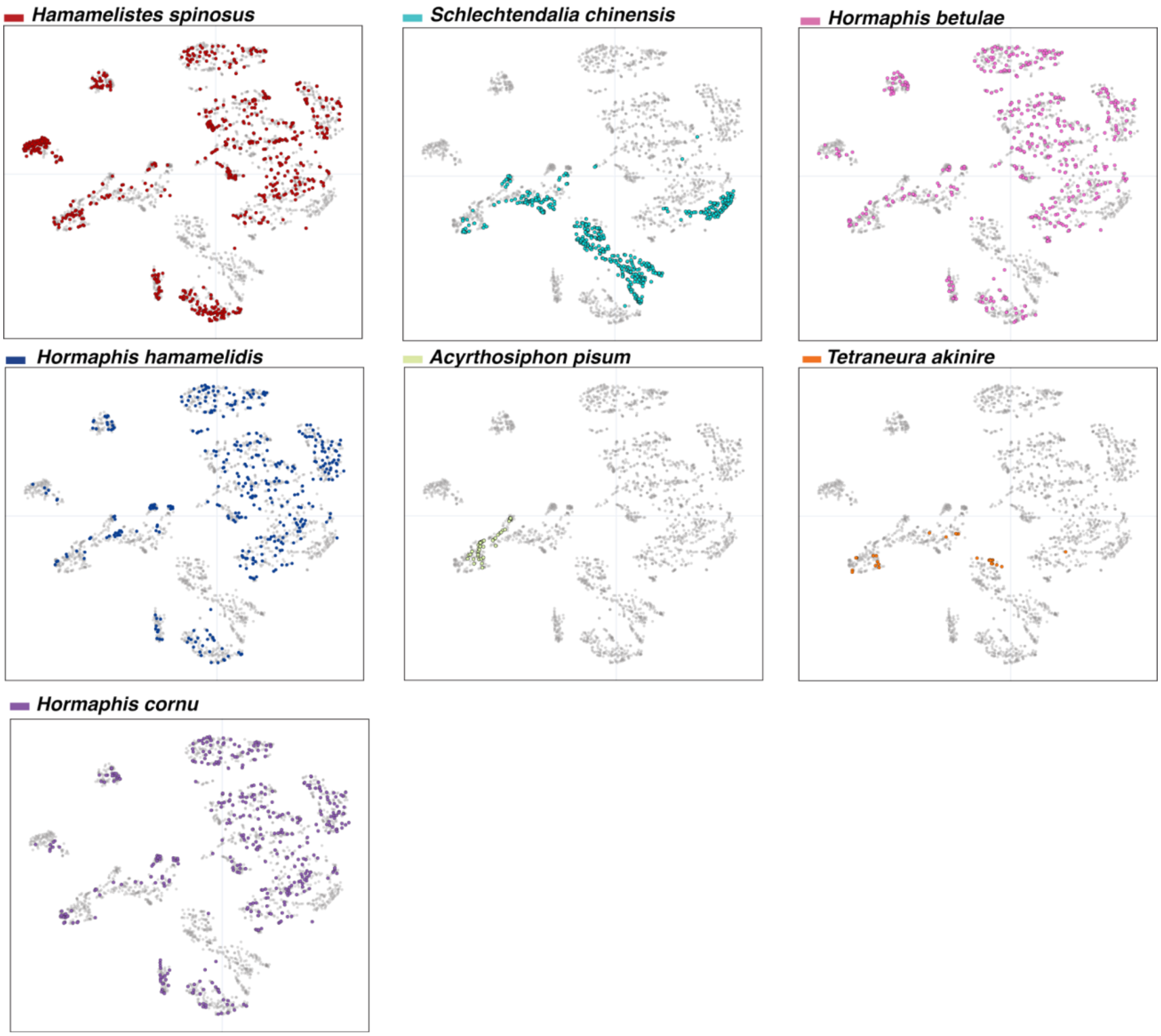

Figure S16: The t-SNE plot of AF2-predicted bicycle protein structures for seven aphid species from Figure 4B, with the species of each protein colored separately in each panel for each of the seven species.

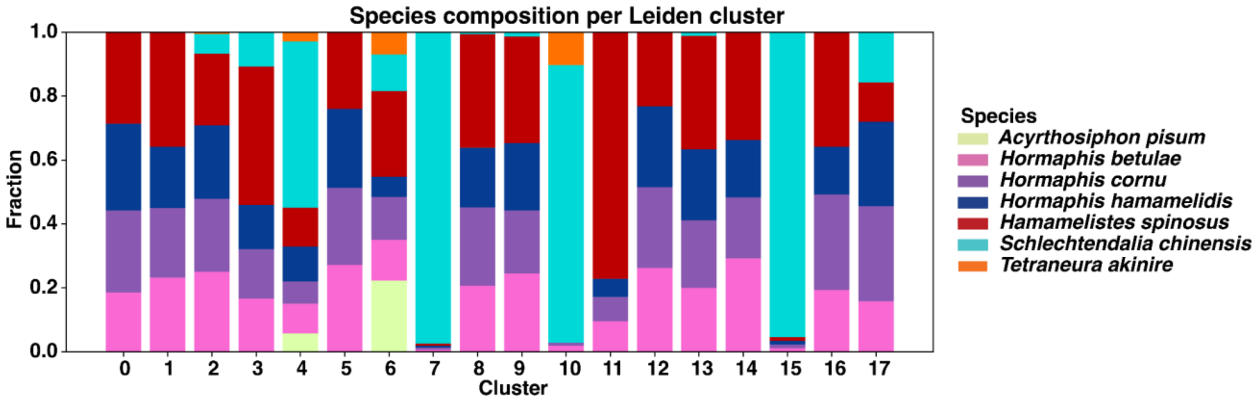

Figure S17: Bar graphs depicting the proportional contribution of proteins from each species to each Leiden cluster in the t-SNE plot from Figure 4B.

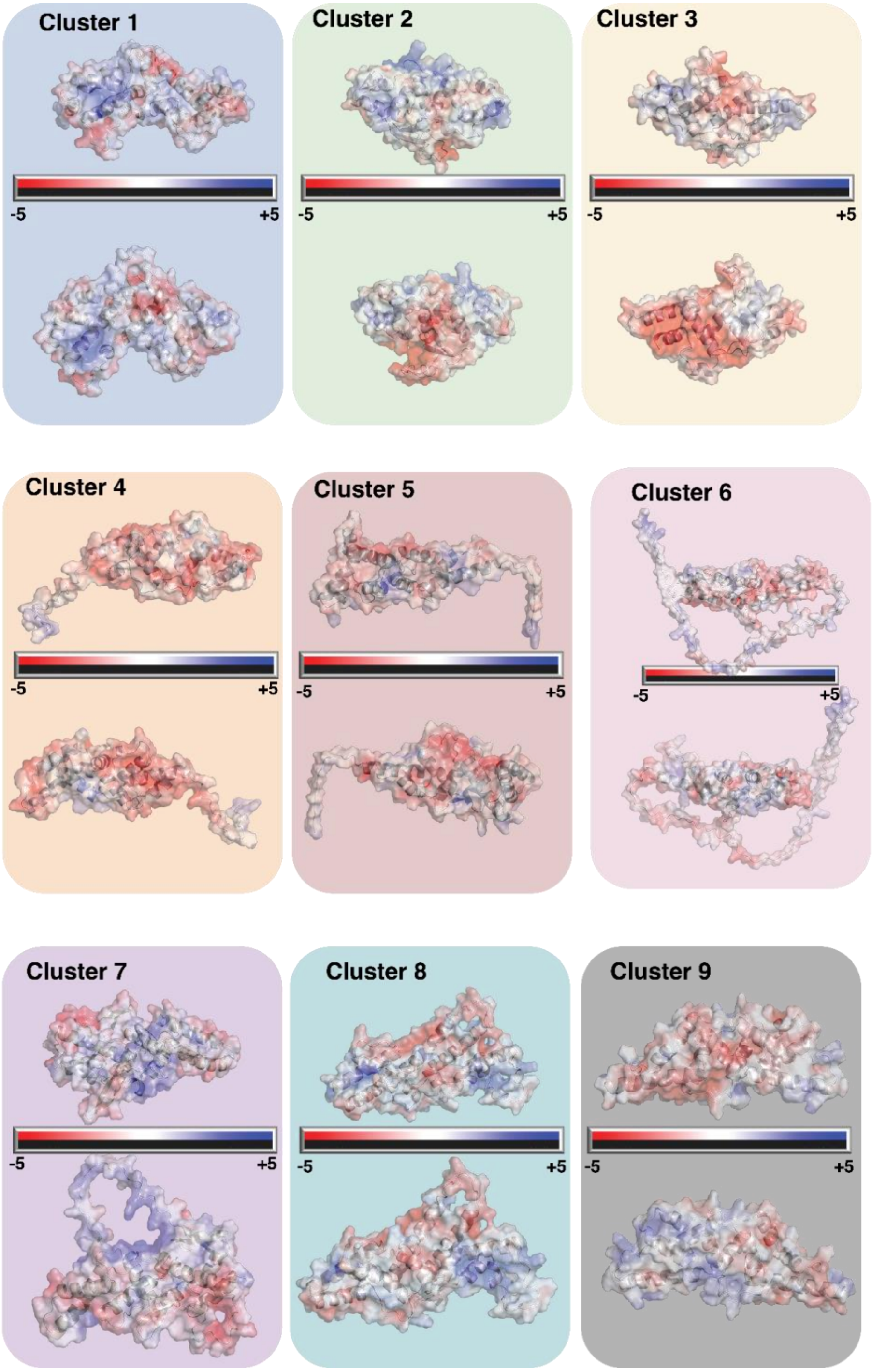

Figure S18: Space filling representation (two views) of APBS electrostatic surface potentials for Leiden cluster medoid models from Figure 5. Electrostatic potentials were computed for the nine cluster medoids using pdb2pqr (AMBER, pH 7.0) and APBS (linearized Poisson–Boltzmann, $\varepsilon_{prot} = 2$, $\varepsilon_{solv} = 78$, 0.15 M monovalent ions). Potentials were mapped onto the molecular surfaces in PyMOL using a fixed color ramp of −5 to +5 kT/e (white ≈ 0).

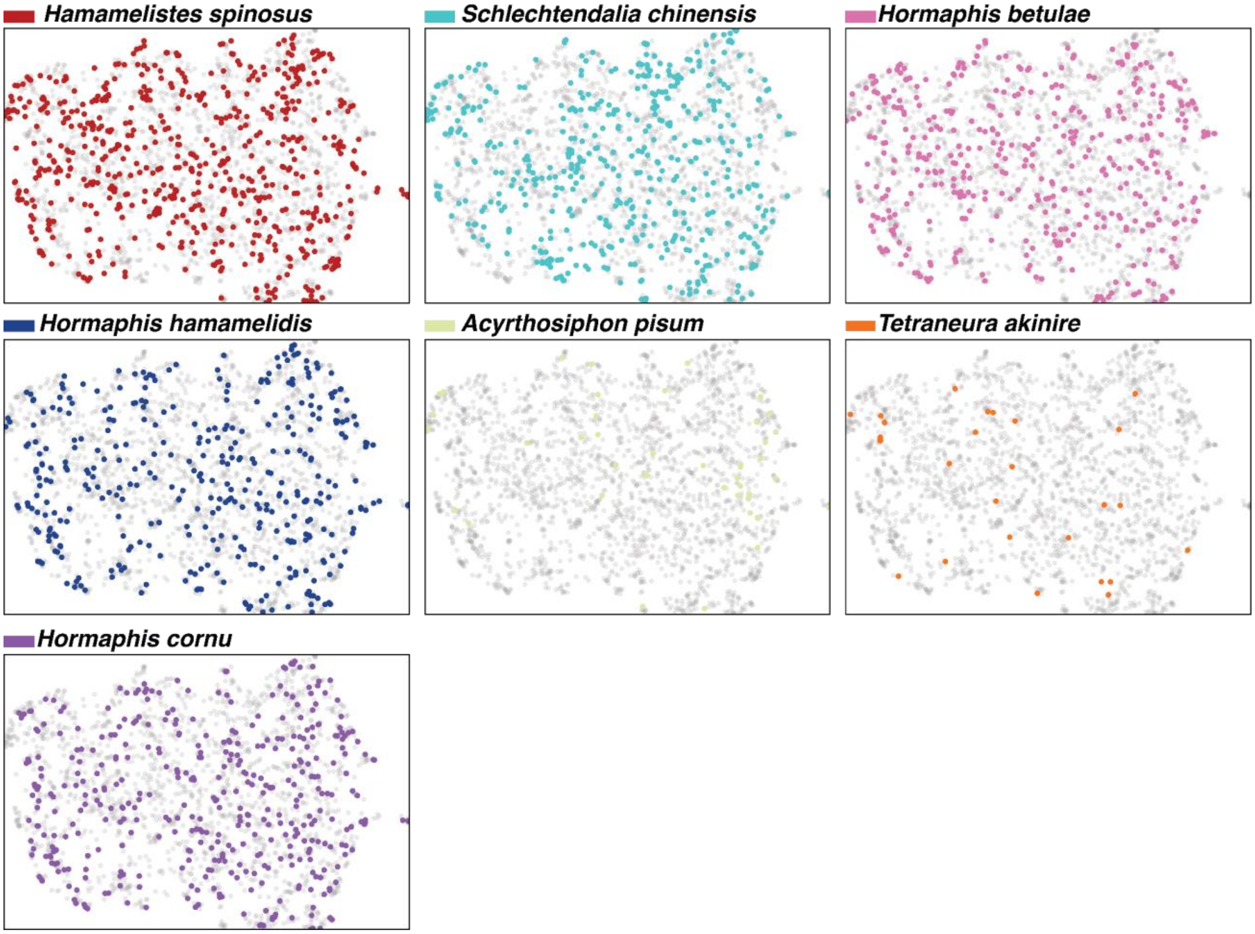

Figure S19: UMAP representation from Figure 5, with the species of each protein colored separately in each panel for each of the seven species.

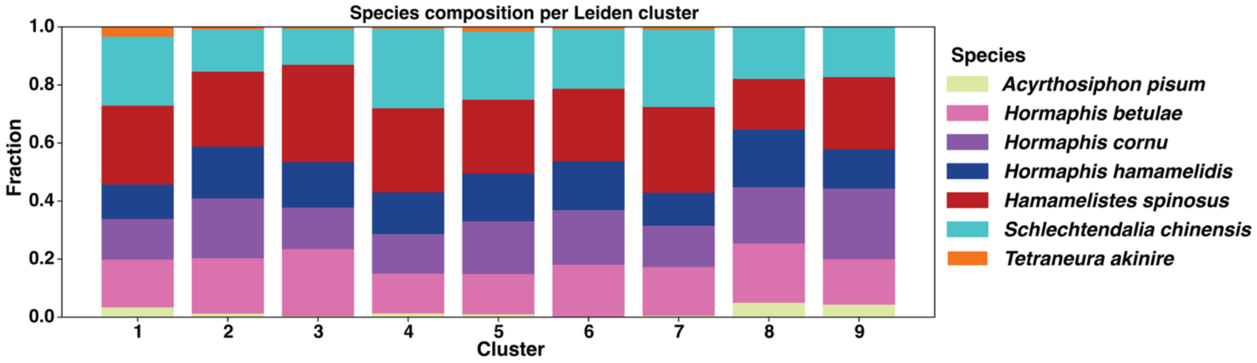

Figure S20: Bar graphs depicting the proportional contribution of proteins from each species to each Leiden cluster in the UMAP plot from Figure 5.

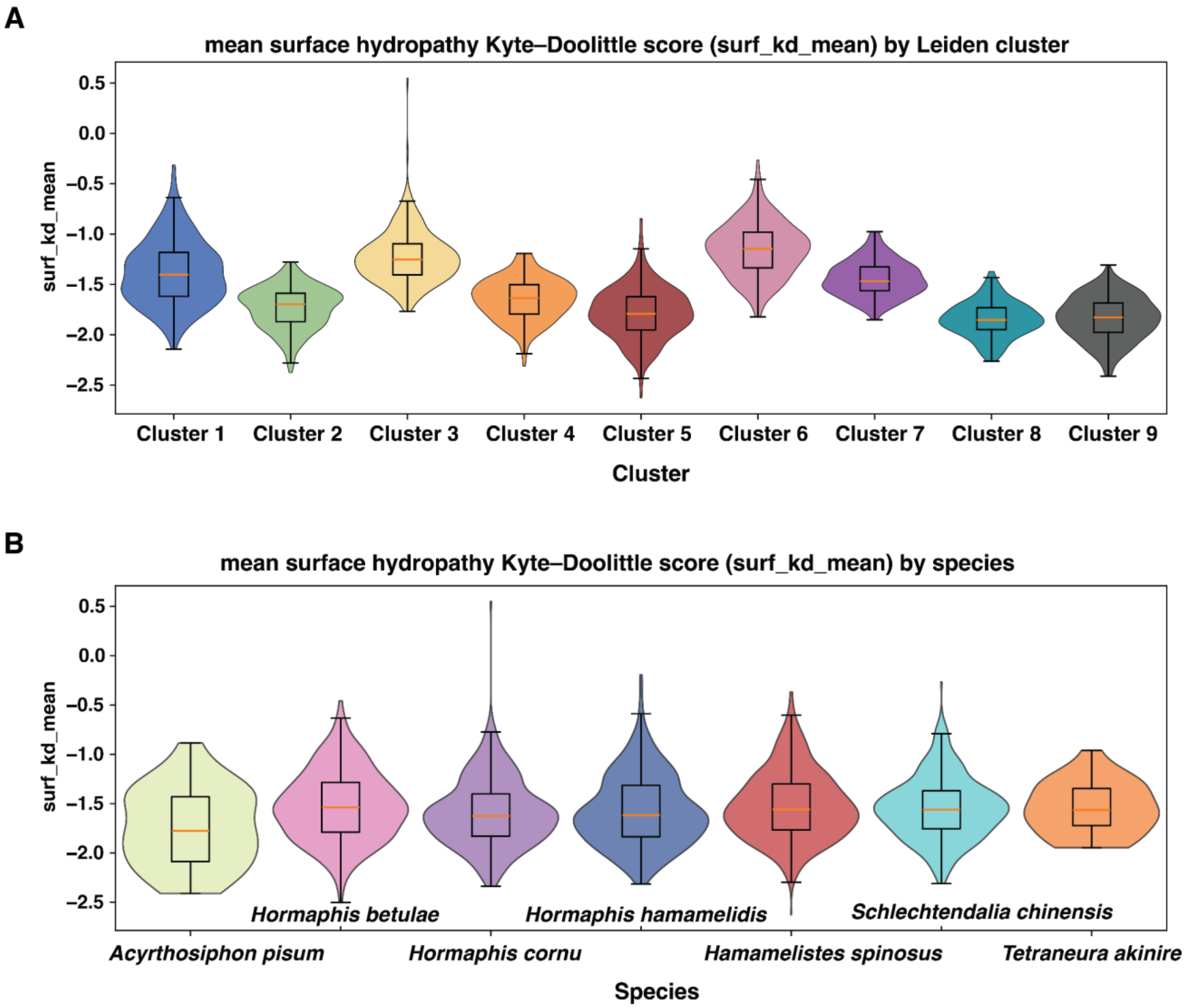

Figure S21: Violin plots of physicochemical property surface hydropathy measured using Kyte-Doolittle score weighted over the solvent exposed surface area by cluster (A) and by species (B).

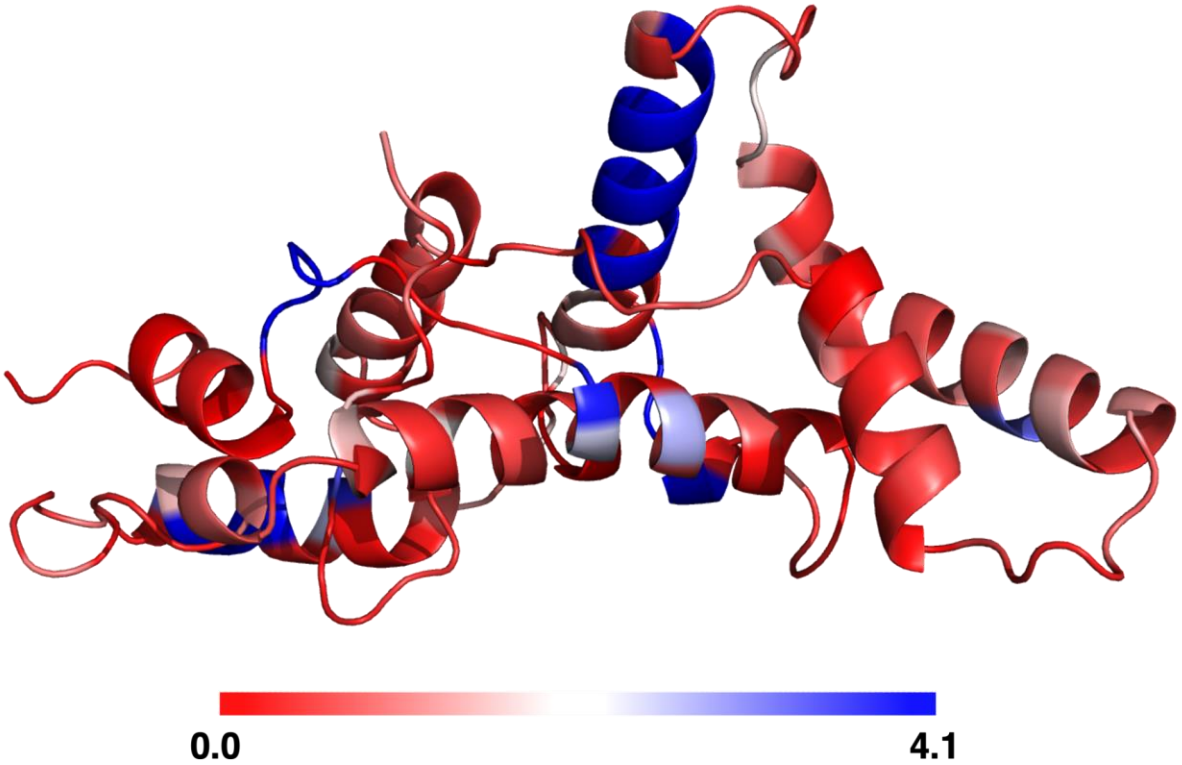

Figure S22: Per-residue sequence variability calculated as Shannon entropy (bits) mapped onto the backbone of the crystal structures of g3873. The ribbon is colored from blue (low entropy = conserved) to red (high entropy = variable) using a fixed scale (0–4.12 bits).

Table S1: Bicycle proteins tested for recombinant expression.

bicycle protein	*E. coli* expression	*SF9* cell expression	Crystals
g3191	No	Yes	Yes
g3873	Yes	Yes	Yes
g7134	No	Yes	No
g15183	No	Yes	No
g16073 (dgc)	Yes	Yes	No
g2703	Yes	Not tested	Yes
g2079	No	Not tested	Not tested
g2080	Yes	Not tested	Not tested
g3028	No	Not tested	Not tested
g3218	No	Not tested	Not tested
g6303	No	Not tested	Not tested
g10552	No	Not tested	Not tested
g11197	No	Not tested	Not tested
g11198	No	Not tested	Not tested
g11199	No	Not tested	Not tested
g13344	No	Not tested	Not tested
g16009	No	Not tested	Not tested
g16059	No	Not tested	Not tested

Table S2: Crystal data collection parameters and structure statistics for g2703 and g3873.

	Se-Meth	Se-Meth	Sulfur-sad	Native
	High-Remote	Low-Remote		
Protein name	g2703		g3873	
PDB ID	9ZML		12AC	
Data Collection Beamline	SSRL-12-1	SSRL-12-1	SSRL-12-1	SSRL-12-1
Space group	P 21 21 21	P 21 21 21	P21 21 21	P21 21 21
Wavelength	0.964	0.984	1.573	0.978
Cell dimensions				
a, b, c (Å)	40.986, 75.996, 85.808	41.002,76.012, 85.827	66.12, 71.268, 193.433	65.98, 71.17, 193.077
alpha, beta, gamma (°)	90.00, 90.00, 90.00	90.00, 90.00, 90.00	90.00, 90.00, 90.00	90.00, 90.00, 90.00
Number of unique reflections	53,411(5,195)	53148(4881)	16045(974)	53700(3568)
Resolution range (Å)	38-1.4	38-1.4	40-3.1	38.9-2.1
Rsym	0.023(0.84)	0.024(0.91)	0.031(0.873)	0.021(0.781)
I / σI	10.8(0.89)	10.9(0.92)	12.7(1.57)	5.5(1.27)
$CC_{1/2}$	0.999(0.273)	0.999(0.269)	0.99(0.371)	0.997(0.291)
Completeness (%)	99.7(98.52)	99.12(92.8)	90.04(94.2)	99.45(98.4)
Redundancy	53.2(43.9)	52.1(36.06)	41.5(39.4)	6.7(6.4)
Refinement				
Resolution (Å)	37.99-1.40			38.96-2.10
Rwork / Rfree	0.1866/0.2053			0.2275/0.2555
No. atoms				
Protein	1452			5374
Ligand/ion	6			-
Water	271			229
B-factors				
Protein	30.41			56.82
Ligand/ion	51.3			-
Water	43.01			51.34
R.m.s. deviations				
Bond lengths (Å)	0.005			0.009
Bond angles (°)	0.835			1.094

Table S3: Disulfide bond parameters in the X-ray structure of G3873.

Chain	Motif	Res 1	Res2	Chi1 (χ 1)	Chi2 (χ 2)	Chi3 (χ 3)	Distance (Å)	Chi2' (χ 2')	Chi1' (χ 1')	Disulfide Strain Energy (kJ/mol)
A	N-terminal Saposin-like (CYC)	30	191	179.89	-89.01	-70.41	2.04	-67.43	-61.91	7.971993
A	non Saposin-like	38	48	-71.09	-136.58	98.06	2.03	-64.94	-75.7	15.178506
A	C-terminal Saposin-like (CYC)	92	120	-74.99	-61.82	-77.47	2.09	-84.16	173.03	8.296798
B	N-terminal Saposin-like (CYC)	30	191	-176.11	-97.13	-76.26	2.05	-74.56	-62.46	9.609778
B	non Saposin-like	38	48	-70.7	-134.93	97.59	2.02	-68.08	-73.45	14.779842
B	C-terminal Saposin-like (CYC)	92	120	-74.11	-61.7	-79.96	2.06	-84.98	171.34	8.278995
C	non Saposin-like	38	48	-55.56	-85.59	-40.94	2.03	90.74	-160.81	29.527321
C	C-terminal Saposin-like (CYC)	92	120	-71.07	-63.7	-75.4	2.07	-83.87	170.17	8.024059
D	N-terminal Saposin-like (CYC)	30	191	-174.79	-88.35	-77.99	2.05	-87.85	-60.28	10.140293
D	non Saposin-like	38	48	-83.74	-135.05	107.05	2.04	-77.57	-80.6	25.848478
D	C-terminal Saposin-like (CYC)	92	120	-74.94	-61.97	-78.27	2.08	-84.62	173.58	8.218305

Table S4: Foldseek hits for g3873 and g2703 identify only poor quality matches (.xlsx file).

Table S5: TM-scores for all saposin-like proteins compared to CYC-domains in g3873 (.xlsx file).

RMSD (Cα matched)	structure	$RMSD = \sqrt{\left(\frac{(1)}{(N)}\right)\sum_{i=1}^{N} d_i^2}$	Å	Higher = less similar; lower RMSD = closer match

Table S6: TM-scores for all saposin-like proteins compared to the sapsin-like CYC-domains in g2703 (.xlsx file).

Table S7: Filtered data of AF2-predicted models for all seven aphid species (.xlsx file).

Table S8: List of physicochemical properties examined for all high-confidence AF2-predicted bicycle proteins.

Column	From	Property	Units	Interpretation
pdb_file	Metadata	ID	—	Identifier
species	Metadata	Species label	—	Identifier
status	Sanity check	QC flag	—	Pass/fail/category
seq_len	Sequence	Length	residues	Larger protein
seq_frac_pos	Sequence	Frac. {K, R, H}	0–1	More basic residues
seq_frac_neg	Sequence	Frac. {D, E}	0–1	More acidic residues
seq_frac_hydrophobic_FLIV	Sequence	Frac. {F, L, I, V}	0–1	More hydrophobic composition
seq_net_charge	Sequence	n(pos) − n(neg)	net residues	More net positive sequence
sasa_total	Structure	Total SASA	$Å^2$	More exposed surface

sasa_hydrophobic	Structure	SASA on Hydrophobic set	Å^2	More hydrophobic surface exposure
sasa_pos	Structure	SASA on Positive set	Å^2	More exposed positive surface
sasa_neg	Structure	SASA on Negative set	Å^2	More exposed negative surface
frac_sasa_hydrophobic[1]	Structure	Hydrophobic surface fraction	0–1	Surface more hydrophobic overall
surf_kd_mean[2]	Sequence and structure	Weighted KD mean (exposed)	KD (unitless)	Exposed surface more hydrophobic on avg
surf_kd_median[3]	Sequence and structure	KD median (exposed)	KD (unitless)	Typical exposed residue more hydrophobic
roughness_sasa_over_hull[4]	Structure	Roughness proxy	unitless	More corrugated/indented surface
hydropatch_count	Structure	No. of hydrophobic patches	count	More fragmented hydrophobic surface
hydropatch_mean_size[5]	Structure	Mean patch size (Σ SASA_res)	Å^2	Larger typical hydrophobic patches
hydropatch_max_size[5]	Structure	Max patch size (Σ SASA_res)	Å^2	One dominant “sticky” patch
surface_charge_dipole[6]	Structure	Charge dipole magnitude |D|	scaled charge·Å	Stronger +/− polarization
exposed_net_charge[7]	Structure	Net exposed charge	net residues	More net positive exposed charge
exposed_net_charge_over_sasa[8]	Structure	Exposed charge density	residues/Å^2	Higher surface charge density
net_charge_over_sasa[8]	Sequence and structure	Seq charge density	residues/Å^2	Higher sequence net charge per area
amphipathic_helix_fraction[9]	Sequence and structure	Amphipathic helix fraction	0–1	More amphipathic helix character
max_hydrophobic_moment[10]	Sequence and structure	Max hydrophobic moment	Unitless	Stronger amphipathic helices

Details:

- Residue sets: Hydrophobic {I, V, L, F, C, M, A, W, Y, P}; Positive {K, R, H partial at pH 7.0}; Negative {D, E}.
- Exposure threshold θ = 5 Å^2.
- Dipole scaling α = 1/100.

- Hydrophobic patches: DBSCAN on exposed hydrophobic sidechain centroids (eps = 7 Å, min_samples = 3), patch size = Σ SASA_res

1. **Fraction hydrophobic SASA:**

$$frac_{sasa_{hydrophobic}} = \frac{(SASA_{hydrophobic})}{(SASA_{total})}$$

2. **SASA-weighted surface KD mean (exposed):**

$$KD_{mean} = \frac{(\Sigma_{exposed} KD(res) \cdot SASA_{res})}{(\Sigma_{exposed} SASA_{res}),\ SASA_{res} \geq \theta}$$

3. **Surface KD median (exposed):**

$$KD_{median} = median\{ KD(res)\} over\ exposed\ residues,\ SASA_{res} \geq \theta$$

4. **Roughness proxy:**

$$roughness = \frac{SASA_{total}}{A_{hull}}$$

5. **Hydrophobic patch size (cluster j):**

$$size_j = \Sigma_{\{res \in cluster\ j\} SASA_{res}}$$

6. **Surface charge dipole magnitude:**

$$D = \Sigma_{exposed} q(res) \cdot (SASA_{res} \cdot \alpha) \cdot r_{centroid(res)};\ \ |D| = \sqrt{Dx^2 + Dy^2 + Dz^2}$$

7. **Exposed net charge:**

$$exposed_{net_{charge}} = \Sigma_{exposed} q(res)$$

8. **Charge densities:**

$$exposed_{net_{charge_{over_{sasa}}}} = \frac{exposed_{net_{charge}}}{SASA_{total}};$$

$$net_{charge_{over_{sasa}}} = \frac{seq_{net_{charge}}}{SASA_{total}}$$

9. **Amphipathic helical fraction:** $\frac{N_{covered}}{L}$

where $N_{covered}$ is the number of residues covered by at least one helical window $\mu_H(i) \geq 0.35$, and L is sequence length, helical mask $h_j = 1$ from $\phi \in [-100°, -30°]$, $\psi \in [-80°, -5°]$ plus min helix run length ≥ 7.

10. **Max hydrophobic moment:**

$$\mu_{H(i)} = \frac{1}{W} \sqrt{\sum_{k=0}^{W-1} H_{i+k}\ cos\ cos\ (k\delta))^2 + (\sum_{k=0}^{W-1} H_{i+k}\ )^2}$$

$$max_{hydrophobic_{moment}} = max_{i:\, h_{i:i+W-1}=1}\ \mu_{H(i)}$$

Table S9: Physicochemical properties of all 2400 bicycle proteins (.xlsx file)

Table S10 : Metric used to generate the overall structure space of proteins

Metric	Derived from	Formula / Definition	Units	Interpretation
TM-score	structure	$TM = \frac{\left(\frac{1}{L_{norm}}\right) sum_{i=1}^{N} 1}{1 + \left(\frac{d_i}{d_0}\right)^2}$ $d_0 = 1.24 * (L_{norm} - 15)^{\frac{1}{3}} - 1.8\ \text{Å}$	unitless (0–1-ish)	Higher = more similar global fold (less sensitive to local outliers than RMSD)

Table S11: Data and statistics of newly assembled and re-annotated genomes (.xlsx file).

References:

1. B. A. Katz, A. Kossiakoff, The crystallographically determined structures of atypical strained disulfides engineered into subtilisin. *J. Biol. Chem.* **261**, 15480–15485 (1986).

2. J. Abramson, *et al.*, Accurate structure prediction of biomolecular interactions with AlphaFold 3. *Nature* **630**, 493–500 (2024).

3. Z. Lin, *et al.*, Evolutionary-scale prediction of atomic-level protein structure with a language model. *Science* **379**, 1123–1130 (2023).

4. M. Blum, *et al.*, InterPro: the protein sequence classification resource in 2025. *Nucleic Acids Res.* **53**, D444–D456 (2025).

5. Y. Zhang, J. Skolnick, TM-align: a protein structure alignment algorithm based on the TM-score. *Nucleic Acids Res.* **33**, 2302–2309 (2005).

6. K. Sakuma, *et al.*, Design of complicated all-α protein structures. *Nat. Struct. Mol. Biol.* **31**, 275–282 (2024).

7. S. Mitternacht, FreeSASA: An open source C library for solvent accessible surface area calculations. *F1000Research* **5**, 189 (2016).

8. P. Virtanen, *et al.*, SciPy 1.0: fundamental algorithms for scientific computing in Python. *Nat. Methods* **17**, 261–272 (2020).

9. N. B. Rego, E. Xi, A. J. Patel, Identifying hydrophobic protein patches to inform protein interaction interfaces. *Proc. Natl. Acad. Sci. U. S. A.* **118**, e2018234118 (2021).

10. V. A. Traag, L. Waltman, N. J. van Eck, From Louvain to Leiden: guaranteeing well-connected communities. *Sci. Rep.* **9**, 5233 (2019).

11. L. van der Maaten, G. Hinton, Visualizing Data using t-SNE. *J. Mach. Learn. Res.* **9**, 2579–2605 (2008).

12. T. J. Dolinsky, *et al.*, PDB2PQR: expanding and upgrading automated preparation of biomolecular structures for molecular simulations. *Nucleic Acids Res.* **35**, W522-525 (2007).

13. E. Jurrus, *et al.*, Improvements to the APBS biomolecular solvation software suite. *Protein Sci. Publ. Protein Soc.* **27**, 112–128 (2018).

14. W. L. DeLano, The PyMOL Molecular Graphics System. (2002). Deposited 2002.

15. O. Gotoh, “Cooperation of Spaln and Prrn5 for Construction of Gene-Structure-Aware Multiple Sequence Alignment” in *Multiple Sequence Alignment*, Methods in Molecular Biology., K. Katoh, Ed. (Springer US, 2021), pp. 71–88.

16. A. Korgaonkar, *et al.*, A novel family of secreted insect proteins linked to plant gall development. *Curr. Biol.* **31**, 1836-1849.e12 (2021).

17. T. J. Booth, S. Shaw, P. Cruz-Morales, T. Weber, getphylo: rapid and automatic generation of multi-locus phylogenetic trees. *BMC Bioinformatics* **26**, 21 (2025).
